# Pre-radiotherapy FDG PET predicts radiation pneumonitis in lung cancer

**DOI:** 10.1186/1748-717X-9-74

**Published:** 2014-03-13

**Authors:** Richard Castillo, Ngoc Pham, Sobiya Ansari, Dmitriy Meshkov, Sarah Castillo, Min Li, Adenike Olanrewaju, Brian Hobbs, Edward Castillo, Thomas Guerrero

**Affiliations:** 1 The University of Texas Health Science Center, Houston, TX, USA; 2 Divisions of Diagnostic Imaging, Houston, TX, USA; 3 Quantitative Sciences, Houston, TX, USA; 4 Radiation Oncology, The University of Texas MD Anderson Cancer Center, Houston, TX, USA; 5 Department of Computational and Applied Mathematics, Rice University, Houston, TX, USA; 6 Baylor College of Medicine, Houston, TX, USA; 7 Department of Radiation Oncology, Unit 97, The University of Texas M. D. Anderson Cancer Center, 1515 Holcombe Blvd, Houston, TX 77030, USA

**Keywords:** Standard uptake value, PET/CT, Radiation pneumonitis, NSCLC, Thoracic radiotherapy, Imaging biomarker

## Abstract

**Background:**

A retrospective analysis is performed to determine if pre-treatment [^18^ F]-2-fluoro-2-deoxyglucose positron emission tomography/computed tomography (FDG PET/CT) image derived parameters can predict radiation pneumonitis (RP) clinical symptoms in lung cancer patients.

**Methods and Materials:**

We retrospectively studied 100 non-small cell lung cancer (NSCLC) patients who underwent FDG PET/CT imaging before initiation of radiotherapy (RT). Pneumonitis symptoms were evaluated using the Common Terminology Criteria for Adverse Events version 4.0 (CTCAEv4) from the consensus of 5 clinicians. Using the cumulative distribution of pre-treatment standard uptake values (SUV) within the lungs, the 80th to 95th percentile SUV values (SUV_80_ to SUV_95_) were determined. The effect of pre-RT FDG uptake, dose, patient and treatment characteristics on pulmonary toxicity was studied using multiple logistic regression.

**Results:**

The study subjects were treated with 3D conformal RT (n = 23), intensity modulated RT (n = 64), and proton therapy (n = 13). Multiple logistic regression analysis demonstrated that elevated pre-RT lung FDG uptake on staging FDG PET was related to development of RP symptoms after RT. A patient of average age and V_30_ with SUV_95_ = 1.5 was an estimated 6.9 times more likely to develop grade ≥ 2 radiation pneumonitis when compared to a patient with SUV_95_ = 0.5 of the same age and identical V_30_. Receiver operating characteristic curve analysis showed the area under the curve was 0.78 (95% CI = 0.69 – 0.87). The CT imaging and dosimetry parameters were found to be poor predictors of RP symptoms.

**Conclusions:**

The pretreatment pulmonary FDG uptake, as quantified by the SUV_95_, predicted symptoms of RP in this study. Elevation in this pre-treatment biomarker identifies a patient group at high risk for post-treatment symptomatic RP.

## Introductions

Radiation pneumonitis (RP), an inflammatory reaction within lung tissue secondary to radiation damage [1,2], is a severe and potentially fatal complication of thoracic radiotherapy (RT). Symptoms of RP include dyspnea, non-productive cough, shortness of breath, fever, and changes in pulmonary function. RP-associated mortality has been noted in the treatment of many cancers including breast [3], esophageal [4,5], lung [6,7], and mesothelioma [8-10]. Furthermore, the mortality rate among non-small cell lung cancer (NSCLC) patients experiencing severe RP symptoms requiring hospitalization approaches 50% [11]. The variability of RP symptoms onset and intensity with respect to patient specific radiation dose, irradiated lung volume, and pulmonary function has made past prognostication efforts futile [12]. Treatment toxicity including RP remains a barrier to radiation dose escalation in lung cancer [13]. Because RP plays such an important role in defining the therapeutic index for lung cancer, clearly there remains a significant need for patient specific prognostication.

Numerous factors such as percentage of lung irradiated [14-16] and chemotherapy type [3,7,17] have been shown to affect occurrence and degree of RP. Another such factor, interstitial pneumonitis (IP) on pretreatment computed tomography (CT) scans, has been shown to predict an increased risk of symptomatic RP [18-20]. Makimoto et al. [18] found that in patients with primary lung cancer, pre-existing lung disease evidenced by pretreatment radiographic changes was associated with a higher incidence of RP (47.1% vs. 5.3%, p < 0.001). Another study showed a correlation between severe RP and pretreatment IP foci in the lung periphery on CT, although exclusion of patients with IP from receiving SBRT led to a reduction in the incidence of severe RP from 18.8% to 3.5% (p = 0.042) in subsequent cases [20]. Additionally, among 106 patients treated with thoracic RT, pretreatment interstitial changes on CT were associated with a higher incidence of grade ≥ 3 RP (26% versus 3%, p < 0.001) [19]. CT scans and x-rays are not the only method to detect pulmonary inflammatory processes. With [^18^F]-2-fluoro-2-deoxyglucose positron emission tomography (FDG PET) imaging, pulmonary inflammation manifests as enhanced FDG uptake, thereby allowing for the quantitative assessment of pneumonitis [21-23]. Recently, Petit et al. [24] performed a retrospective study of 101 NSCLC patients to evaluate the correlation between symptomatic RP and pre-RT FDG PET/CT evidence of pulmonary inflammation. They report that the 95th percentile of the standard uptake value (SUV_95_) within the lungs was predictive of RP on multivariate analysis (p = 0.016), suggesting that the SUV_95_ can be used to screen for RP risk during thoracic RT treatment planning [24].

In this retrospective study, pre-RT FDG PET/CT image derived factors are analyzed as potential prognostic biomarkers of symptomatic RP in NSCLC patients, testing the findings reported by Petit et al. [24]. We hypothesize that these pre-RT image derived factors identify individuals at high risk for symptomatic RP.

## Methods and Materials

### Patient population

The study population consisted of 100 non-small cell lung cancer patients who were treated in the Department of Radiation Oncology at the University of Texas M. D. Anderson Cancer Center between July 2004 and May 2012, and who had their staging PET/CT imaging within 90 days prior to the start of radiotherapy. All study subjects had biopsy-proven NSCLC, and their imaging studies are available in the electronic medical records. Patient characteristics were obtained for each study subject including age, sex, disease stage, tumor location, smoking history, tumor histologic type, radiation planning, interval between staging PET and RT, concurrent chemotherapy, and pre-existing lung disease (as assessed by FEV1 and DLCO parameters). Patient identifiers were removed in accordance with a retrospective study protocol (PA11-0801) approved by the MD Anderson Institutional Review Board. Waiver of informed consent was approved by the Institutional Review Board for this retrospective study protocol.

### ^18^F-FDG PET/CT imaging

Patients fasted 6 hours prior to the ^18^F-FDG PET/CT imaging session and were required to have blood glucose levels < 120 mg/dL. Intravenous injection of 629 (range: 550 – 740) MBq of ^18^F-FDG occurred 60 (range: 52–110) minutes prior the image acquisition. The General Electric Discovery ST PET/CT scanner (GE Medical Systems, Waukesha, WI) was used to acquire the ^18^F-FDG PET/CT images. Patients were instructed to breath normally during the PET emission acquisition. The ^18^F-FDG PET images included in this study acquired before 2006 were attenuation corrected using a non-contrast mid-inspiratory breath-hold CT, and those after used a respiratory averaged CT [25]. PET/CT images were acquired from mid-thigh to the skull base with arms raised. Standard uptake values (SUV) were calculated from the attenuation corrected ^18^F-FDG PET emission images using the following equation [26]:

(1) $$\text{Standard}\;\text{Uptake}\;\text{Value}=\frac{{}^{18}F‒\text{FGD}\;\text{count}\;\text{rate}\;\text{per}\;\mathrm{mL}\times \text{body}\;\text{weight}\left(\mathrm{gm}\right)}{\text{decay}\;\text{correcte}d^{18\;}F‒\text{FDG}\;\text{injected}\;\text{dose}\;\left(\mathrm{Bq}\right)}$$

### Radiation treatment planning

Treatment planning for megavoltage x-ray cases was performed using the Pinnacle^3^ version 7.6c or 8.0u treatment-planning system (Philips Medical Systems, Andover, MA). Proton therapy cases were planned using the respiratory averaged CT and the Eclipse treatment planning system (Varian Medical Systems, Palo Alto, CA). Gross target delineation and margin generation were performed in a consistent manner, as previously reported by our group [27]. Radiation dose was calculated using either free-breathing treatment planning CT data (most cases) or averaged CT data obtained from the treatment planning 4D CT image set [28,29]. All treatment plans and field arrangements were prospectively reviewed in quality assurance meetings in which consensus was obtained according to each patient's clinical circumstances. The radiation dose distributions were all calculated using lung heterogeneity corrections. The mean lung dose (MLD) and the percentage of lung volume irradiated to above 5 Gy or CGE (V_5_), 10 Gy or CGE (V_10_), 20 Gy or CGE (V_20_), and 30 Gy or CGE (V_30_) were used as dosimetric parameters to represent the lung volumes irradiated.

For proton cases, all plans were designed for passive scattering delivery. Using a constant relative biological effectiveness (RBE) of 1.1, proton therapy doses were converted to ^60^Co Gray Equivalents (CGE).

### Clinical Toxicity and Radiation Parameters

Pneumonitis was scored using the National Cancer Institute Common Terminology Criteria for Adverse Events version 4 (CTCAE v4). All patient documents were used in the scoring, including consultation notes, radiographic images, clinic notes, summaries and scanned outside medical records until 6 months after completing radiation. A simple group consensus of 5 clinicians was used for each score. Cases were reviewed until all discrepancies were resolved by unanimous agreement. Clinically symptomatic pneumonitis was defined as grade 2 or higher. All patients with RP scores > 1 had radiographic findings consistent with RP within the radiotherapy treatment field. These findings were evident on follow-up CT imaging and/or PET/CT.

### Image analysis

**Figure 1:**
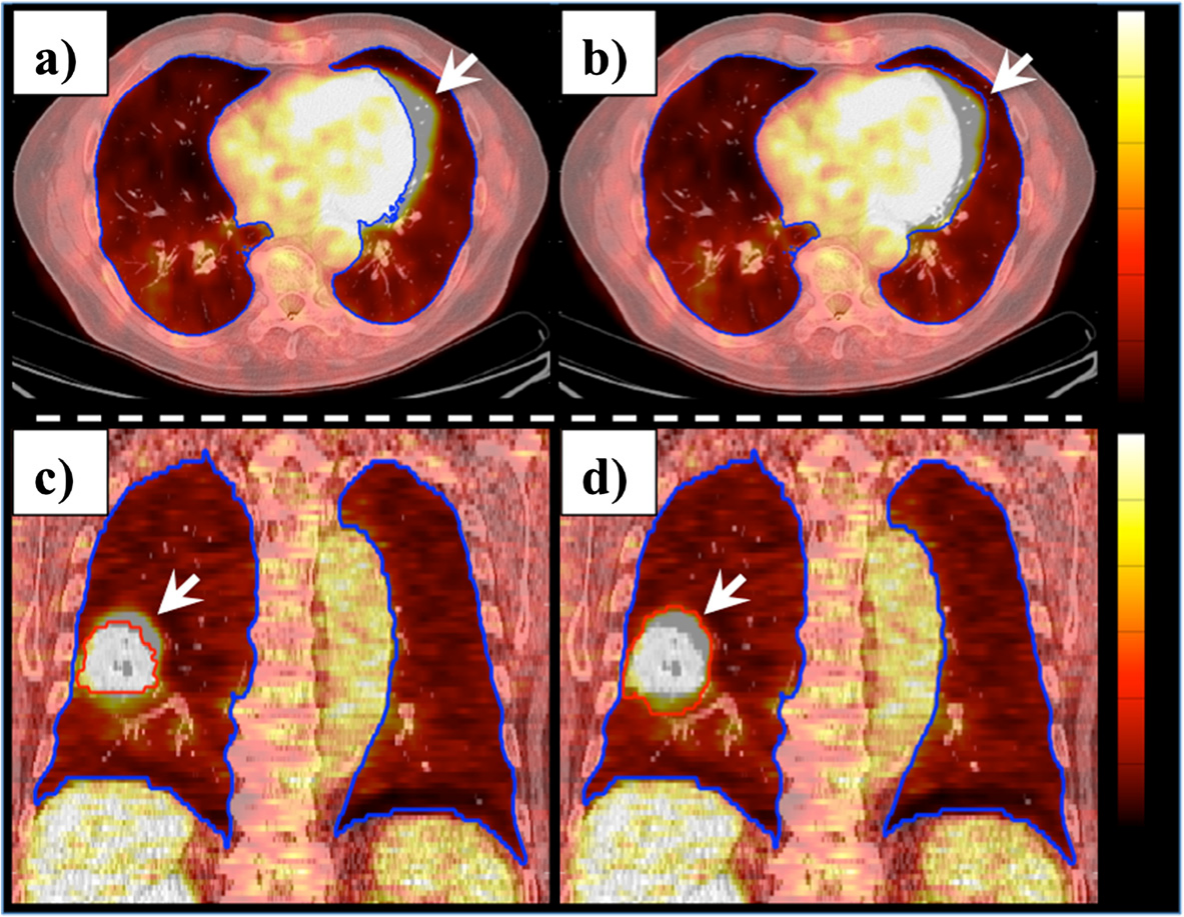
**Lung segmentation and removal of PET spill-over activity artifacts.** Semi-automated histogram segmentation and morphological region growing were used to delineate the set of lung voxels on the pre-treatment staging PET/CT studies. PET spill-over activities into the lung ROI were manually contoured for exclusion. *Top row*: lung ROI shown **(a)** before and **(b)** after manual correction of cardiac spill-over activity. *Bottom row*: the region of exclusion due to tumor is show in red **(c)** before and **(d)** after manual correction.

The treatment plan and PET/CT images for each patient were processed and evaluated using custom MATLAB software (v2011a, Mathworks, Inc.). Lung regions of interest (ROIs) were segmented semi-automatically using histogram segmentation of the lung parenchyma and removal of the central airway by connectivity. PET spill-over artifacts (Figure 1) attributable to liver, heart, or tumor activities were manually contoured for exclusion from the segmented lung volume. Attenuation cold-spot artifacts at the diaphragm surface [30] were also manually removed. The effect of manual editing on the lung ROI and subsequent analysis was assessed according to repeat image segmentation performed by 3 independent secondary reviewers in a subsample of 10 patients (10% of all cases). The primary reader binary lung ROI was used in subsequent analyses.

### Pretreatment PET/CT analysis

**Figure 2:**
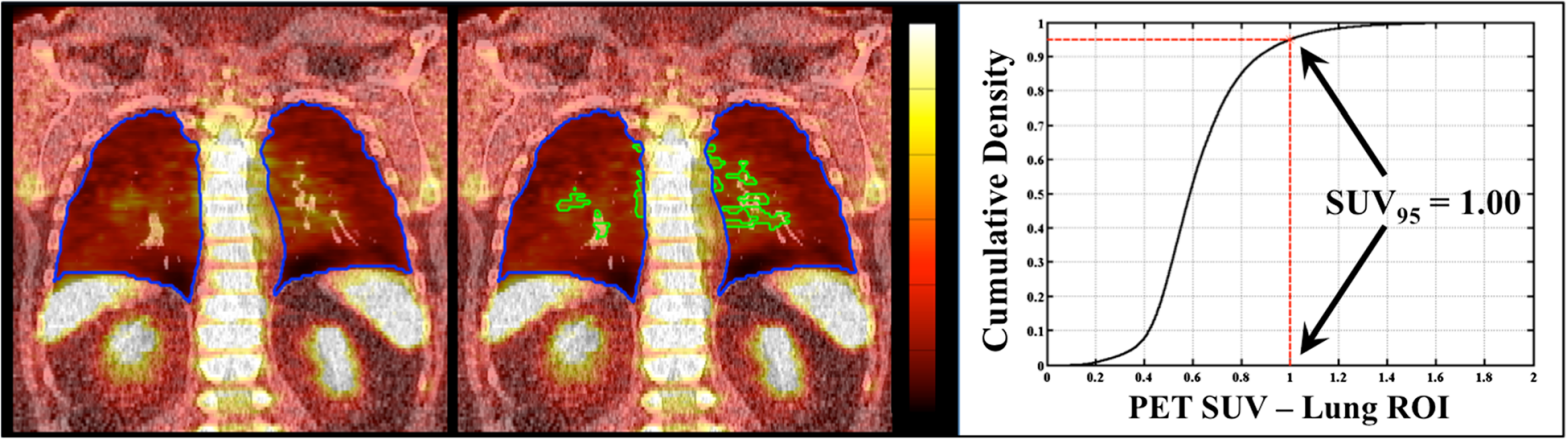
**Quantifying the SUV**_**95**_**.** SUV values within the lung are determined by semi-automated segmentation of the lung voxels from the pre-treatment staging PET/CT study (*left*). The cumulative distribution of SUV values is constructed from the voxel values within the lung ROI. The 80th, 90th, and 95th percentiles are obtained from these distributions to yield the corresponding SUV_80_, SUV_90_, and SUV_95_. The SUV_95_ is depicted graphically (*right*) for the example case, with the ROI ≥ SUV_95_ shown superimposed (*middle*).

Using the pretreatment FDG PET images, the SUV of all voxels within the lung ROI were binned into histograms, and the mean SUV (SUV_mean_), the standard deviation of the SUV (SUV_SD_), and the maximum SUV (SUV_max_) were calculated as described in Petit et al. [24]. A cumulative probability distribution was constructed from each histogram (Figure 2) and used to determine the 80th, 90th, and 95th percentiles of the SUV distribution, hereafter designated: SUV_80_, SUV_90_, and SUV_95_, respectively. To determine if pre-treatment CT density could predict RP, the cumulative density parameters mentioned above were also calculated for Hounsfield Unit (HU) of the CT scan: the HU_mean_, HU_SD_, HU_max_, HU_80_, HU_90_, and HU_95_.

### Statistical Analysis

Categorical variables (i.e., gender, tumor stage, tumor location, tumor histologic type, radiotherapy modality, chemotherapy status, smoking status, GOLD classification) were summarized using frequency tables; evaluated for association with symptomatic (grade ≥ 2) RP using Pearson’s chi-squared test for marginal homogeneity. Age and the interval between radiotherapy and PET imaging were summarized by median and range; evaluated for association with symptomatic (grade ≥ 2) radiation pneumonitis using Mann–Whitney U tests. Univariate logistic regression analyses were used to predict symptomatic (grade ≥ 2) RP as functions of pre-RT pulmonary and dosimetry characteristics (i.e., SUV, HU, MLD, irradiated volume, FEV1%, DLCO%). Post-hoc application of the sequentially rejective Bonferroni method [31] was used to adjust for multiplicity among the six SUV analyses.

Multiple logistic regression inference used stepwise backward model selection based on Akaike information criterion [32]. Results are provided for the best subset of predictors (SUV_95_, V_30_, age). Partial effects were evaluated for significance using two-sided Wald tests. Nagelkerke’s coefficient of multiple determination [33] is used to report the proportion reduction in error variation obtained by incorporating the predictors. The resultant receiver operating characteristic (ROC) curve is provided with Delong’s 95% confidence interval [34] for the area under the curve (AUC) and Youden’s optimal [35] specificity and sensitivity. Additionally, recursive partitioning analysis [36] was used to formulate a binary classification tree based upon both SUV_95_ and V_30_. Kaplan-Meier curves were used to compare time to radiation pneumonitis symptom development among the observed terciles of SUV_95_ (SUV_95_ < 0.99, 0.99 ≤ SUV_95_ < 1.2, SUV_95_ ≥ 1.2); Cox proportional hazard regression was used to evaluate the rate of RP symptom development as a function of SUV_95_ adjusted for patient and treatment characteristics. Stepwise backward model selection used generalized Akaike information criterion [29]. Results are provided for the best subset of predictors (SUV_95_, V_30_, age). Inter-reviewer variability in determination of SUV_95_ was assessed for 3 independent reviewers in a subsample of 10 patients; 95% limits of agreement were estimated using one-way mixed effects ANOVA [37]. The resultant Bland-Altman plot [38] is provided. All tests were two sided with α = 0.05 to confer statistical significance. All plots and analyses were performed using the statistical software R (R Development Core Team, http://www.r-project.org) version 3.0.

## Results

### Patient Characteristics and RP Symptoms

An overview of the 100 study subjects and their characteristics is presented in Tables 1 and 2. Of the study participants, 14 (14%) were treated with RT alone while 86 (86%) received concurrent chemo-radiation (chemoRT). The prescription dose range was 36 to 74 Gy (median 66) over 12–37 fractions (median 35). The mean lung dose was between 2.88 and 29.43 Gy (median 17.86 Gy). Consensus CTCAEv4 RP symptom scores were: 10 patients (10%) had no evidence of respiratory symptoms or imaging changes (grade 0), 31 patients (31%) had only radiographic or mild respiratory symptoms without requirement of intervention (grade 1), 27 patients (27%) had post-RT respiratory symptoms affecting the extended activities of daily living (grade 2), 23 (23%) required oxygen (grade 3), 1 (1%) respiratory failure requiring intubation (grade 4) and, 8 (8%) died from respiratory compromise (grade 5). A total of 60% of the patients experienced symptomatic RP.

**Table 1:** Patient Characteristics

**Parameter**	**Total (%)**	**Symptomatic**^**a **^**N (%)**	**p-value**
No of patients	100 (100)	59 (59)	
**Gender**			0.65
Male/Female	60(60)/40(40)	35(58.3)/26(65)	
**Age**			**0.011**
Symptomatic (IQR)		59.5-73 **yrs**	
Asymptomatic (IQR)		54-66 **yrs**	
**Stage**^b^			0.36
I	6 (6)	1 (16.7)	
II	5 (5)	4 (80)	
III	78 (78)	48 (61.5)	
IV	11 (11)	6 (54.5)	
**Tumor location**^b^			0.97
LLL	15 (15)	10 (66.7)	
LUL	25 (25)	15 (60)	
RLL	9 (9)	6 (66.7)	
RML	6 (6)	4 (66.7)	
RUL	45 (45)	24 (53.3)	
**Tumor histology**^b^			0.66
Adenocarcinoma	57 (57)	30 (52.6)	
Neuroendocrine	1 (1)	1 (100)	
Non-small	18 (18)	13 (72.2)	
Squamous	24 (24)	15 (62.5)	
**Treatment type**^b^			0.89
IMRT	64 (64)	35 (54.7)	
Proton	13 (13)	9 (69.2)	
3D Conformal	23 (23)	15 (65.2)	
**Chemotherapy status**			0.66
Concurrent	86 (86)	52 (60.5)	
RT alone	14 (14)	7 (50)	
**Smoking history**^b^			0.92
Currently	28 (28)	15 (53.6)	
Former	66 (66)	40 (60.6)	
Never	6 (6)	4 (66.7)	
**Interval between staging PET/CT and start of RT**			0.65
Median (range) in days	18 (3–69)	15 (3–69)	

^a^Symptomatic status: CTCAEv4 RP grade ≥ 2.

^b^Yates’ continuity correction applied.

IQR: Inter-quartile range.

*Note*: Hypothesis testing for association used the Mann–Whitney *U* test for continuous predictors; Pearson’s chi-squared test for marginal homogeneity for categorical predictors.

**Table 2:** Treatment characteristics and outcomes

**Treatment dose (Gy or CGE)**	
Range (median)	36 – 74 (66)
Mean lung dose (Gy or CGE)	
Range (median)	2.88 – 29.43 (17.86)
^a^Radiation pneumonitis symptom score, n (%)	
0	10 (10)
1	31 (31)
2	27 (27)
3	23 (23)
4	1 (1)
5	8 (8)
Pre-RT pulmonary function test, range (median)	
FEV_1_ (%)	30 – 124 (72.5)
DLCO (%)	23 – 125 (64)

^a^Symptomatic status: CTCAEv4 RP grade ≥ 2.

The patient demographics, stage, tumor location, tumor histology, treatment type and smoking history are reported in Table 1 for the total and symptomatic (CTCAEv4 RP grade ≥ 2). Treatment characteristics and outcomes are listed in Table 2. The data lacked significant evidence to conclude that the presence of symptomatic RT was associated with other clinical factors including tumor stage, histology, location, type of RT, or preexisting lung disease based on FEV1 parameters, as well as any CT-derived imaging parameters.

### PreRT SUV_95_, V_30_ and age predict for radiation pneumonitis

Age was the only non-modifying factor found to be significantly associated with the development of symptomatic RP using the Mann–Whitney U hypothesis test. Univariate logistic regression analyses are summarized in Table 3. Odds of grade ≥ 2 radiation pneumonitis increased with SUV_mean_, SUV_SD_, SUV_80_, SUV_90_, and SUV_95_ as well as V_30_. SUV_95_ was the most significant independent predictor of post-radiation lung toxicity (p < 0.0049). In addition, significant partial effects were observed for SUV_95_ (p < 0.0027), V_30_ (p < 0.007), and age (p < 0.0026) in the multiple logistic regression analysis provided in Table 4. For a given age and value of V_30_, each incremental increase in SUV_95_ of size 0.1 was associated with a 1.5-fold increase (95% CI: 1.1 – 1.9, p < 0.0027) in the partial odds of symptomatic RP. A patient of average age (64) and V_30_ (23.8) with a value of SUV_95_ = 1.2 (1.5) is 1.4 (6.9) times more likely to develop symptomatic RP when compared to a patient presenting with SUV_95_ = 1 (0.5) of the same age and identical V_30_. Additionally, the partial odds of symptomatic RP increased 2.2-fold with each increase in age of 1 year and 1.1-fold with each unit increase in V_30_, respectively.

**Table 3:** Logistic regression analysis for grade ≥ 2 RP

**Predictor**	**Coefficient**	**SE**	**Odds ratio (95% CI)**	**p-value**
SUV_max_	0.16	0.095	1.2 (0.97, 1.4)	0.10
SUV_mean_	0.34	0.15	1.4 (1.1, 1.9)	**<0.02**^**a,b**^
SUV_SD_^1^	1.5	0.54	4.4 (1.5, 12.8)	**<0.0057**^**a,b**^
SUV_80_^1^	0.31	0.13	1.4 (1.1, 1.7)	**<0.013**^**a,b**^
SUV_90_^1^	0.33	0.12	1.4 (1.1, 1.7)	**<0.0061**^**a,b**^
SUV_95_^1^	0.33	0.12	1.4 (1.1, 1.7)	**<0.0049**^**a,b**^
HU_max_^2^	0.013	0.02	1 (0.97, 1.1)	0.51
HU_mean_^2^	-0.09	0.34	0.91 (0.47, 1.8)	0.79
HU_sd_^2^	-0.18	0.93	0.83 (0.14, 5.1)	0.85
HU_80_^2^	-0.07	0.37	0.93 (0.46, 1.9)	0.85
HU_90_^2^	-0.10	0.32	0.91 (0.48, 1.7)	0.76
HU_95_^2^	-0.10	0.32	0.91 (0.48, 1.7)	0.76
MLD^3^	0.88	0.58	2.4 (0.78, 7.5)	0.13
V_5_^3^	0.15	0.53	1.2 (0.41, 3.3)	0.77
V_10_^3^	0.58	0.55	1.8 (0.6, 5.3)	0.30
V_20_^3^	1.1	0.63	3 (0.87, 10.5)	0.081
V_30_^3^	1.2	0.57	3.3 (1.1, 10.3)	**0.035**
FEV1(%)^2^	-0.13	1	0.88 (0.12, 6.6)	0.897
DLCO(%)^2^	-1.2	1.1	0.31 (0.034, 2.9)	0.31

^1^Scaled by 10.

^2^Scaled by 0.01.

^3^Log-transformation applied to improve model fit.

^a^Sequentially rejective Bonferroni method applied to adjust for multiplicity at the α = 0.05 familywise significance level.

^b^Odds of grade ≥ 2 radiation pneumonitis increased with SUV_mean_, SUV_SD_, SUV_80_, SUV_90_, and SUV_95_.

*Note*: SE = standard error of the estimated coefficient parameter; CI = confidence interval for the odds ratio; p-values derived from two-sided hypothesis tests using Wald chi-square.

**Table 4:** Multiple logistic regression analysis for grade ≥ 2 RP (N = 100)

**Predictor**	**Coefficient**	**SE**	**Odds ratio (95% CI)**	**p-value**
Intercept	-6.1	1.7		
SUV_95_^a^	0.40	0.13	1.5 (1.1-1.9)	**<0.0027**
Age^b^	0.79	0.26	2.2 (1.3-3.7)	**<0.0026**
V_30_	0.09	0.034	1.1 (1–1.2)	**<0.007**

^a^Scaled by 10.

^b^Standardized age was used with origin corresponding to the mean of 64.

*Note*: SE = standard error of the estimated coefficient parameter; CI = confidence interval for the odds ratio; Stepwise backward model selection based on Akaike information criterion was used; Symptomatic radiation pneumonitis was conditionally independent of tumor location, stage, histology, smoking status, MLD, and RT modality in the presence of SUV_95_, V_30_, and age; p-values derived from two-sided hypothesis tests using Wald chi-square; significant partial effects suggest that the odds of symptomatic radiation pneumonitis increased with SUV_95_, V_30_, and age; Nagelkerke coefficient of multiple determination R^2^ = 0.32.

**Figure 3:**
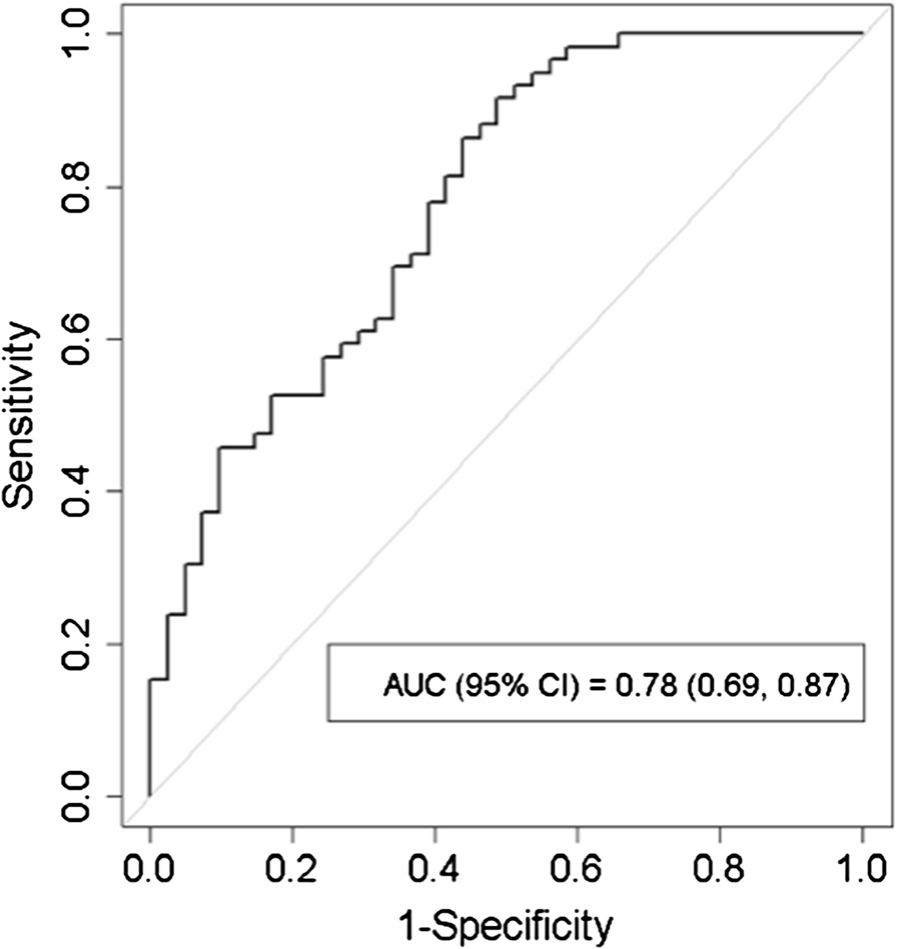
**Receiver operating characteristics curve for RP symptoms.** Receiver Operating Characteristic (ROC) curve (solid) derived from pre-treatment SUV_95_, V_30_, and age to predict symptomatic (grade ≥ 2) radiation pneumonitis. The area under the ROC curve derived from the multiple logistic regression inference was found to be 0.78 (95% CI: 0.69 – 0.87) with Youden’s optimal sensitivity = 92% and specificity = 51%.

**Figure 4:**
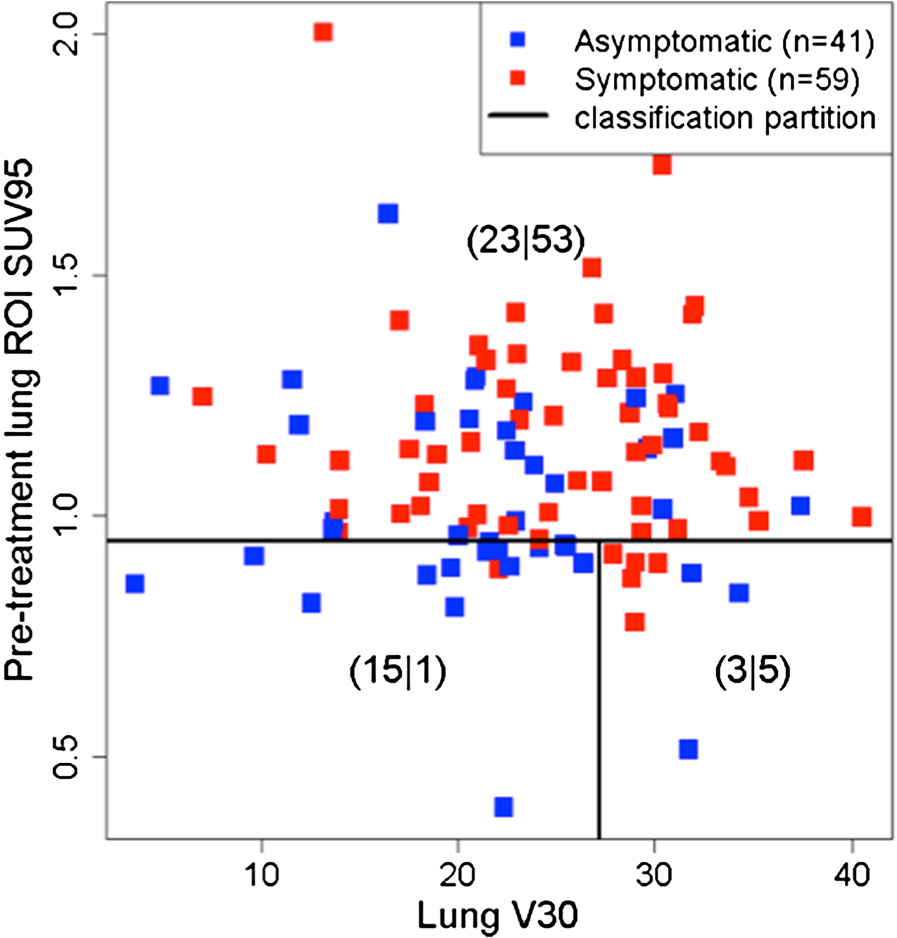
**Classification of symptomatic and asymptomatic RP against SUV**_**95 **_**and V**_**30**_**.** Recursive partition analysis for classification of RP symptoms using pre-treatment SUV_95_ and V_30_ for N = 100 lung cancer patients results in 3 cohorts. The optimal partition derives from classifying patients with pre-treatment SUV_95_ > 0.949 or V_30_ > 27.14 as symptomatic, and those with SUV_95_ < 0.949 and V_30_ < 27.14 as asymptomatic. The joint classification tree results in sensitivity = 98% and specificity = 37%.

Receiver Operating Characteristic (ROC) analysis derived from pre-treatment SUV_95_, V_30_, and age to predict symptomatic (grade ≥ 2) radiation pneumonitis is shown in Figure 3. The area under the ROC curve derived from the multiple logistic regression inference was found to be 0.78 (95% CI = 0.69 – 0.87) with Youden’s optimal sensitivity = 92% and specificity = 51%. The distribution of symptomatic and asymptomatic is plotted against SUV_95_ and V_30_ in Figure 4. Recursive partition analysis for classification of RP symptoms using pre-treatment SUV_95_ and V_30_ in 3 cohorts is also shown. The optimal partition (assuming identical misclassification costs) derives from classifying patients with pre-treatment SUV_95_ > 0.949 or V_30_ > 27.14 as symptomatic, patients with SUV_95_ < 0.949 and V_30_ < 27.14 as asymptomatic. The joint classification tree results in sensitivity = 98% and specificity = 37%.

### SUV_95_ Influences Time to Development of Radiation Pneumonitis

**Figure 5:**
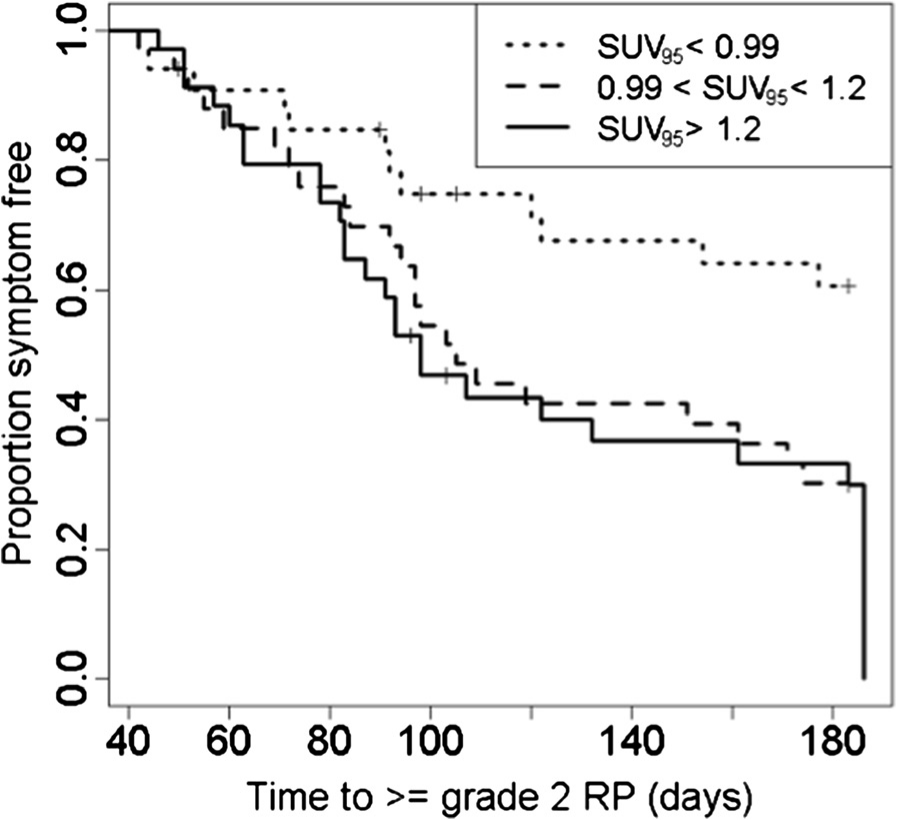
**Kaplan-Meier curves for pre-treatment SUV**_**95**_**.** Time to radiation pneumonitis symptoms is compared among subsets of patients within observed terciles of SUV_95_ (SUV_95_ < 0.99, 0.99 ≤ SUV_95_ < 1.2, SUV_95_ ≥ 1.2). Right-censored observations are marked by +. The hazard ratios (HR) and corresponding 95% confidence intervals for comparing between the (2nd and 1st) terciles; and (3rd and 1st) terciles follow as 2.25 (1.12, 4.52) and 2.39 (1.19, 4.82), respectively. Median time to symptoms for patients with SUV_95_ ≥ 0.99 was 101 days.

Among patients who developed symptomatic RP, the average time from start of RT to symptomatic development was observed to be 3.5 months for patients with SUV_95_ > 0.99 and 4.5 months for patients with SUV_95_ < 0.99. Kaplan-Meier curves were constructed to compare time to radiation pneumonitis symptoms among subsets of patients within observed terciles of SUV_95_ (SUV_95_ < 0.99, 0.99 ≤ SUV_95_ < 1.2, SUV_95_ ≥ 1.2). Figure 5 shows that patients with SUV_95_ ≥ 1.2 developed symptoms at a rate 2.39 (1.19, 4.82) times the rate of patients with SUV_95_ < 0.99, while patients with 0.99 ≤ SUV_95_ < 1.2 developed symptoms at a rate 2.25 (1.12, 4.52) times greater.

Additionally, multiple Cox proportional hazards regression was used to evaluate the association between SUV_95_ and time to development of symptomatic RP, adjusted for age and V_30_ (Table 5). The odds of developing symptomatic RP within a given duration of time increased with SUV_95_, age, and V_30_. SUV_95_ contributed the most significant partial effect (p < 0.002). Given age and V_30_, each incremental 0.1 increase in SUV_95_ was associated with a 1.2-fold increase (1.1, 1.3) in the partial hazard rate of RP symptom development.

**Table 5:** Cox proportional hazards regression analysis for time to radiation pneumonitis symptoms (N = 100)

**Predictor**	**Coefficient**	**SE**	**Hazard ratio (95% CI)**	**p-value**
SUV_95_^a^	0.18	0.057	1.2 (1.1-1.3)	**<0.002**
Age^b^	0.34	0.14	1.4 (1.1-1.8)	**0.013**
V_30_	0.05	0.02	1.1 (1–1.1)	**0.011**

^a^Scaled by 10.

^b^Standardized age was used with origin corresponding to the mean of 64.

*Note*: SE = standard error of the estimated coefficient parameter; CI = confidence interval for the hazard ratio; Stepwise backward model selection based on generalized Akaike information criterion was used; symptomatic radiation pneumonitis was conditionally independent of tumor location, stage, histology, smoking status, treatment type, and MLD in the presence of SUV_95_, V_30_, and age; p-values derived from two-sided hypothesis tests using Wald chi-square; the rate of symptom development was increased significantly with SUV_95_, V_30_, and age.

### Inter-reviewer agreement for acquisition of SUV_95_

**Figure 6:**
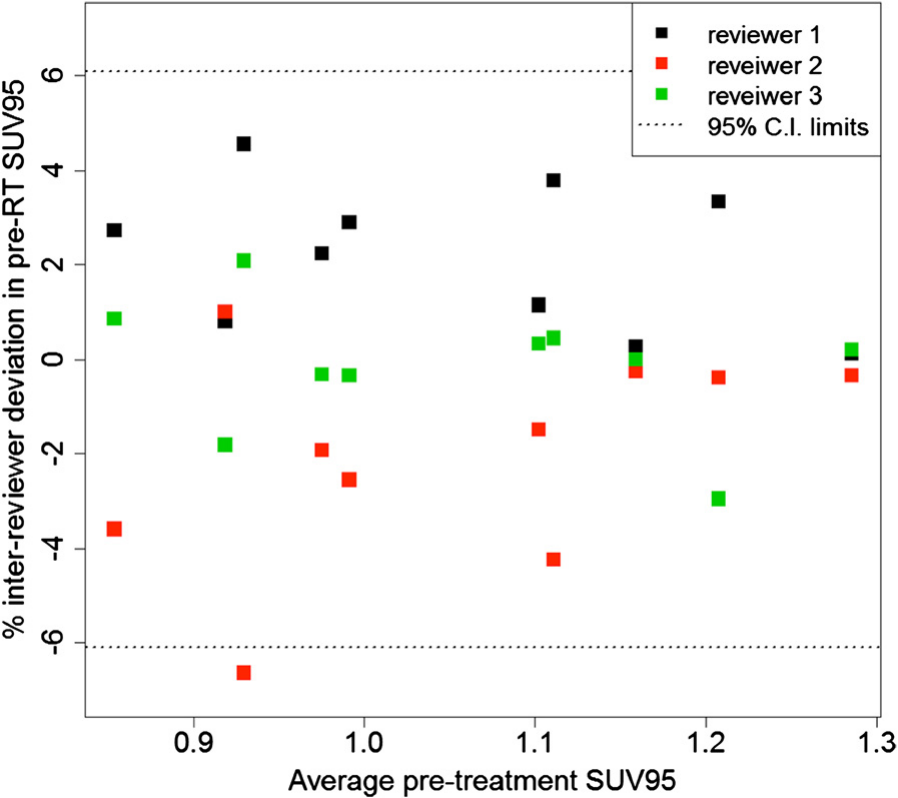
**Bland-Altman plot for inter-reviewer agreement in the determination of pre-treatment lung SUV**_**95**_**.** Observed and expected percentage deviation from mean SUV_95_ in a subsample of 10 patients assessed by three independent reviewers. One-way mixed effects ANOVA obtains 95% confidence boundaries = ±6.10%.

Inter-reviewer agreement among three independent reviewers for determination of SUV_95_ using a representative 10% of all cases (10 subsampled patients) is plotted in Figure 6. Inter-reviewer deviation was within approximately 6% of the reviewer average at the α = 0.05 significance level. The observed variation among reviewers reflects the inherent subjectivity associated with the manual intervention to remove PET spill-over activity artifacts (Figure 1) and SUV cold spot artifacts at the lung/diaphragm interface due to respiration. While deviation on the order of 6% is not innocuous given the magnitude of association between the risk of RP and the pre-treatment SUV_95_, this represents the 95% limit of agreement based upon a subset of 10 patients. Thus we expect on average that inter-reader deviation would be on the order of ± 3%, which corresponds to only a 0.88 to 1.12-fold change in the odds of symptomatic RP.

## Discussion

In this study, we demonstrated the potential of a quantitative image derived prognostic biomarker, the SUV_95_, for the pre-treatment identification of NSCLC patients at high risk to develop symptomatic RP. This biomarker provides a quantitative assessment of pre-existing pulmonary inflammation [22,39], which in turn predicts the individual subject’s ability to tolerate thoracic radiation without toxicity. This study, which includes a mixture of proton and photon treated lung cancer cases, replicates the finding of Petit et al. [24] who studied a photon-only treated NSCLC cohort. Dehing et al. [40] previously analyzed data from a photon-only treated cohort of 438 patients with NSCLC or SCLC to assess predictive value of patient characteristics and dosimetric parameters associated with dyspnea following thoracic chemo-radiotherapy. Univariate models with V_20_ (mean: 21%, SD: 7.3%) or MLD (mean: 13.5 Gy, SD: 4.5 Gy) both yielded AUC of 0.47. The final multivariate model, which included WHO-performance status, smoking status, forced expiratory volume, age, and MLD, yielded an AUC of 0.62 (95% CI: 0.55-0.69). However, the authors cite that baseline dyspnea scores were not available to rule out the possibility that patients with low FEV1 values already had an elevated dyspnea score prior to treatment. The current study supports the previous findings by Dehing et al. that a combination of patient-related factors and dosimetric parameters, namely the SUV_95_, V_30_, and age, is better suited as a prognostic indicator for symptomatic outcomes following thoracic radiotherapy. Pretreatment FDG PET/CT imaging is already routinely obtained for staging of NSCLC [41-43] and has an emerging role in target delineation for radiotherapy treatment planning for NSCLC [44,45]. The SUV_95_, computed from imaging studies already obtained for staging and treatment planning, can be used to stratify toxicity risk without incurring additional cost.

Notably, the significant association between Hounsfield Unit derived parameters and increase in dyspnea reported by Petit et al. [24] did not hold in the current analysis. The difference may arise due to the difference in CT acquisition methods between studies. Although Petit et al. describe both respiratory gated 4D-CT and low-dose CT with intravenous contrast for each patient, it is not clear which CT image set was utilized to calculate the lung region of interest (ROI) Hounsfield Unit values. In this study, Hounsfield Unit ROI parameters were derived utilizing the radiotherapy treatment planning CT, which was a mix of either free-breathing CT (FB-CT) or 4D-CT.

Other imaging modalities have been utilized to estimate the pretreatment symptomatic RP risk. The relationship between the radiation dose distribution and subsequent RP has been well studied and is summarized nicely by Rodrigues et al. [6]. Single photon emission computed tomography (SPECT) perfusion imaging has been utilized to demonstrate radiation-induced lung toxicity [46,47], showing a nearly linear loss of perfusion with radiation dose. Kocak et al. [12] prospectively tested RP prediction models based on pulmonary perfusion and radiation dose distributions using models built from one data set and tested on two other data sets. Those models were unable to segregate patients into high and low risk of RP groups in the test data sets. Others have utilized pretreatment ventilation imaging to predict RP in single cohort retrospective studies [48]; however the ROC AUC was small. Hope et al. [49] developed a 3-parameter model (from the tumor superior-inferior relative position, maximum dose, and dose to the hottest 35% of the lung volume), which was tested using a separate data set (RTOG 9311) by Bradley et al. [50] and performed poorly. The STRIPE meta-analysis of pneumonitis after chemoradiotherapy for lung cancer [7] found that concurrent paclitaxel, age, and V_20_ were significant predictive factors with odds ratios of 5.58, 1.38, and 1.07 respectively. Paclitaxel is a radiosensitizer of lung tissue [3,51] that can cause pneumonitis even when used alone [52-54]. The SUV_95_ quantifies pre-existing pulmonary inflammation, the severity of which may reflect the underlying individual propensity toward an inflammatory response.

For lung cancer clinical trials involving thoracic radiation with pulmonary toxicity as an end-point, the SUV_95_ can be utilized to (1) ensure equally balanced arms or (2) exclude those who appear to have a nearly 100% certainty of developing symptomatic pulmonary toxicity. An analysis of a prospective clinical trial conducted by the Radiotherapy Oncology Group (RTOG) indicates higher biologically effective doses of radiotherapy are associated with improved outcomes [55]. However, the recently completed prospective study RTOG 0617 found no advantage as well as increased toxicity in the higher dose arm [13]. Biomarkers such as the SUV_95_ may be used for stratification to enroll only low RP risk study subjects. The SUV_95_ can also be utilized to identify a subgroup at high risk for the development of RP symptoms for clinical trials studying RP-prevention drugs. A cohort with an expected high incidence of RP would power a drug RP prevention trial using fewer study subjects to measure a reduction in RP toxicity events.

Our study was limited by the retrospective nature of this analysis, which could contain inherent biases that we are not aware of despite our best efforts to control for potential confounders. The 3D-CRT patients were treated in an earlier time period, which may have accounted for increased toxicities with less modern imaging and treatment planning techniques. Additionally, the 3D PET images were not acquired with motion correlation [56], thus contributing to spatial blurring and spill-over activity artifacts that required manual intervention processes to exclude from data analysis. Pneumonitis grade was scored using the medical record rather than standardized questionnaires. A prospective study addressing the pulmonary toxicity should include standardized survey such as the St. George Respiratory Questionnaire [57].

## Conclusions

In the present study, patients with high FDG uptake prior to treatment were more likely to develop symptomatic RP. Our findings may be used to identify patients at high risk for radiation-induced lung damage so that interventions can be developed and fatal RP avoided.

## Abbreviations

RP: Radiation pneumonitis; RT: Radiotherapy; NSCLC: Non-small cell lung cancer; CT: Computed tomography; IP: Interstitial pneumonitis; FDG: ^18^ F-2-fluoro-deoxyglucose; PET: Positron emission tomography; SUV: Standard uptake value; MLD: Mean lung dose; CGE: ^60^Co Gray Equivalents; CTCAE v4: National Cancer Institute Common Terminology Criteria for Adverse Events version 4; ROI: Region of interest; HU: Hounsfield Unit; ROC: Receiver operating characteristic; AUC: Area under the curve; RTOG: Radiotherapy oncology group.

## Competing interests

The authors have no commercial or financial interests related to this study to disclose.

## Authors’ contribution

RC contributed to study conception and design, data analysis, and drafting of the manuscript. NP, SA, and DM contributed to data analysis and drafting of the manuscript. SC and AO contributed to data acquisition processes and data analysis. ML and EC contributed to the development of data analysis infrastructure, with further contribution to data analysis processes. BH performed statistical testing and contributed to drafting of the manuscript. TG formulated study conception and design, and contributed to drafting of the manuscript. All authors provided final approval of the manuscript version to be published.
